# Selecting the right patients is the key

**DOI:** 10.7196/AJTCCM2018.v24i1.204

**Published:** 2018-04-03

**Authors:** Richard van Zyl-Smit, Greg Calligaro

**Affiliations:** 1 Lung Clinical Research Unit, University of Cape Town Lung Institute and Division of Pulmonology, Department of Medicine, University of Cape Town and Groote Schuur Hospital, Cape Town, South Africa; 2 Division of Pulmonology, Department of Medicine, University of Cape Town and Groote Schuur Hospital, Cape Town, South Africa

## Editorial

The pre-operative evaluation of patients undergoing resectional lung surgery is an integral part of assessing the risk of perioperative complications, and estimating the likelihood of sufficient postoperative pulmonary reserve.^[1]^ In emergency situations, such as massive haemoptysis, a full evaluation is not possible and judgement is based on an assessment of pre-morbid effort tolerance and on the radiological and lung function parameters available at the time. For elective surgery, there is the opportunity to perform both resting and dynamic investigations to fully evaluate patients and to identify those with suspected inadequate postoperative pulmonary reserve.^[2,3]^

In this issue of *AJTCCM*, Amirali *et al*. ^[4]^ compare forced expiratory volume in 1 second (FEV_1_ ), transfer factor (DLCO) and aerobic capacity (VO_2_ max) in two groups of patients: those undergoing resection for lung cancer, and those with post-inflammatory lung disease. The lung cancer patients had higher average pre-operative FEV_1_ values (62% of predicted v. 52%), which translated (based on expected lung to be removed) into a higher predicted post-operative FEV_1_ (41% v. 34%). There was no difference in the DLCO values between the groups; however, the predicted postoperative VO_2_ max was also higher in the inflammatory lung disease group.

The authors suggest that the lung cancer patients would have a better pre-operative functional reserve. The clinical applicability of this descriptive report is limited, as neither postoperative mortality nor complications were recorded in the two groups.

This study highlights one important aspect of evaluation of patients undergoing lung surgery; however, there are many additional aspects that need to be taken into account over and above physiology. The age of patients and co-morbid conditions are critical factors influencing postoperative recovery and risk for complication – even simple smoking status affects outcomes and wound healing.^[4,5]^ The experience of the surgeon, the quality of the intensive care, as well as postoperative nutrition, wound care and physiotherapy all affect the outcomes, and although the predicted postoperative functional capacity may be more than adequate, any disruption to these other parameters may result in poor outcomes – not related to the predicted physiology.^[6]^

All patients undergoing major surgery, including pulmonary resection – especially those who have borderline respiratory functional reserve, significant co-morbidities and potential to not withstand any complications – should be carefully evaluated. A functional assessment may support the decision either to opt for surgery when the physiology would suggest they will withstand resection, or to support the decision to not operate when the physiology suggests lack of reserve, despite other parameters being favourable. Physiological evaluation is the cornerstone as postoperative predictive equations will allow robust evaluations of patients who would not tolerate lung resection.^[7]^

In these groups of patients, a multi-disciplinary approach involving all the stakeholders, including physicians, surgeons, anaesthetists, intensivists, the patient and their family members is required. The alternatives are potentially poor, unpredicted and fatal outcomes. Given the high risks involved, patient involvement is critical in the decision-making process and adequate family counselling of what to expect, what the potential risks are and how the decision to go ahead or not was made, will ensure that all potential outcomes are foreseen and planned for.
